# A structural approach to the assessment of fracture risk in children and adolescents with chronic kidney disease

**DOI:** 10.1007/s00467-007-0490-6

**Published:** 2007-07-11

**Authors:** Mary B. Leonard

**Affiliations:** Departments of Pediatric and Epidemiology, University of Pennsylvania School of Medicine, The Children’s Hospital of Philadelphia, 34th Street and Civic Center Blvd, CHOP North, Room 1564, Philadelphia, PA 19104 USA

**Keywords:** Renal osteodystrophy, Fracture, DXA, Bone mineral density, Bone

## Abstract

Children with chronic kidney disease (CKD) have multiple risk factors for impaired accretion of trabecular and cortical bone. CKD during childhood poses an immediate fracture risk and compromises adult bone mass, resulting in significantly greater skeletal fragility throughout life. High-turnover disease initially results in thickened trabeculae, with greater bone volume. As disease progresses, resorption cavities dissect trabeculae, connectivity degrades, and bone volume decreases. Increased bone turnover also results in increased cortical porosity and decreased cortical thickness. Dual-energy X-ray absorptiometry (DXA)-based measures of bone mineral density (BMD) are derived from the total bone mass within the projected bone area (g/cm^2^), concealing distinct disease effects in trabecular and cortical bone. In contrast, peripheral quantitative computed tomography (pQCT) estimates volumetric BMD (vBMD, g/cm^3^), distinguishes between cortical and trabecular bone, and provides accurate estimates of cortical dimensions. Recent data have confirmed that pQCT measures of cortical vBMD and thickness provide substantially greater fracture discrimination in adult dialysis patients compared with hip or spine DXA. The following review considers the structural effects of renal osteodystrophy as it relates to fracture risk and the potential advantages and disadvantages of DXA and alternative measures of bone density, geometry, and microarchitecture, such as pQCT, micro-CT (μCT), and micro magnetic resonance imaging (μMRI) for fracture risk assessment.

## Introduction

Renal osteodystrophy is a multifactorial and universal disorder of bone metabolism in chronic kidney disease (CKD). As renal failure progresses, ensuing abnormal parathyroid hormone (PTH) secretion results in deterioration of trabecular microarchitecture, thinning of cortical bone, and increased cortical porosity [1]. Despite widespread use of phosphate binders and vitamin D therapies, hip fracture rates in young adults on dialysis are 100-fold greater than in healthy controls [2]. Successful renal transplantation corrects many of the underlying abnormalities contributing to renal osteodystrophy. However, persistent hyperparathyroidism, immunosuppressive therapies, and renal allograft dysfunction may impair recovery of renal osteodystrophy and lead to further bone loss. Hip fracture rates among adult dialysis patients undergoing renal transplantation increase even further in the early months following transplantation, then gradually decline [3]. Significantly greater vertebral and extremity fracture rates have also been demonstrated in pediatric solid organ transplant recipients compared with healthy controls: the age- and sex- adjusted hazard ratios for vertebral fractures were 61.3 [95% confidence interval (CI), 40.7–92.4) compared with over 200,000 population-based controls [4].

Throughout childhood and adolescence, normal bone mineral acquisition results in gender-, maturation-, and site-specific increases in bone density and dimensions. Children with CKD have multiple risk factors for impaired bone development, including abnormal mineral metabolism, secondary hyperparathyroidism, poor linear growth, delayed development, malnutrition (including vitamin D insufficiency), decreased weight-bearing activity, and immunosuppressive therapies. The impact of these threats to bone health may be immediate, resulting in fragility fractures, or delayed, due to suboptimal peak bone mass accrual. The signs and symptoms of renal bone disease seen in adults, such as subperiosteal resorption, osteosclerosis, fractures and muscle weakness, may also complicate CKD in childhood. In addition, the effects of abnormal bone and mineral metabolism on endochondral ossification during growth result in complications in the epiphyseal region that are unique to children with CKD. These complications include linear growth failure, slipped epiphyses, and skeletal deformities resembling vitamin-D-deficient rickets. Back pain, scoliosis, and narrowed, degenerated disc spaces also complicate renal transplantation in childhood [5].

The majority of studies of bone loss in CKD relied on dual-energy X-ray absorptiometry (DXA) measures of bone mineral density (BMD). However, DXA summarizes the total bone mass within the projected bone area (g/cm^2^), concealing distinct structural alterations in trabecular and cortical bone density and architecture. DXA has additional important limitations in children and adolescents, such as sparse reference data and lack of consensus regarding techniques to adjust for the confounding effect of bone size, as described by us [6] and others [7] in this journal. The following review considers the structural effects of renal osteodystrophy as it relates to fracture risk and the potential advantages and disadvantages of DXA and alternative measures of bone density, geometry, and microarchitecture, such as peripheral quantitative computed tomography (pQCT), micro-CT (μCT), and micro magnetic resonance imaging (μMRI) for fracture risk assessment.

## Histomorphometry of renal osteodystrophy

Renal osteodystrophy is an early, pervasive. and multifactorial disorder of bone remodeling that often develops prior to any clinical manifestations of CKD [1]. The classic form of renal osteodystrophy is high-turnover disease due to secondary hyperparathyroidism. Phosphate retention, hypocalcemia, reduced renal 1α-hydroxylation of 25(OH) vitamin D to generate 1,25(OH)_2_ vitamin D, and skeletal resistance to the actions of PTH contribute to the marked hyperparathyroidism in CKD. Therapies to increase serum calcium, decrease serum phosphate, and reduce PTH levels have resulted in decreased frequency and severity of hyperparathyroidism. However, the incidence of low-turnover disease (adynamic bone disease) has subsequently increased.

Whereas early case series of bone biopsy results in children on maintenance dialysis reported high-turnover bone disease (osteitis fibrosa and mild lesions of secondary hyperparathyroidism) in the vast majority of patients [8–10], more recent series identified adynamic bone in a substantial proportion (27–33%) of children and adolescents [11–13]. The implications of low bone turnover for bone structure and strength during growth are not known; however, this bone lesion is associated with increased fracture risk in adults [14] — perhaps due to impaired microfracture repair. It has been suggested that intermittent calcitriol therapy combined with calcium-containing phosphate binders may adversely affect chondrocyte activity in the epiphyseal growth plate, with a consequent reduction in linear growth. Studies on the relations between adynamic bone, suppression of PTH with vitamin D therapies, and growth in children have yielded conflicting results [15–17].

The trabecular manifestations of renal osteodystrophy are well-described in iliac crest bone biopsies [18]. The increased remodeling rate in high-turnover disease initially results in thickened, irregularly shaped trabeculae with increased bone volume [19]. Increased bone volume is not necessarily equated with greater bone strength because the irregular trabeculae may lose their proper three-dimensional (3D) architecture and connectivity. As disease progresses, resorption cavities dissect trabeculae, connectivity degrades, and bone volume decreases. Low-turnover disease is associated with decreased bone volume and variable alterations in trabecular architecture.

Traditionally, reports of bone biopsies in patients with renal osteodystrophy focused on trabecular morphology to the exclusion of cortical bone. However, hyperparathyroidism may increase subperiosteal, endosteal, and intracortical resorption, resulting in cortical bone loss on multiple surfaces [1]. The catabolic effect of increased PTH on cortical bone structure is primarily characterized by increased cortical porosity and decreased cortical thickness. A study of full-thickness bone biopsies in adult dialysis patients demonstrated a 45% reduction in cortical bone volume in both high-turnover and low-turnover disease [19].

Although I am unaware of any reports of cortical bone porosity or dimensions on bone biopsies in children with CKD, pQCT studies of cortical bone in children and adolescents confirm significant deficits in density and dimensions. Lima et al. reported that cortical volumetric bone mineral density (vBMD, g/cm^3^) was significantly lower in children with CKD compared with controls [20]. Ruth et al. reported that cortical thickness was significantly lower in the proximal and distal radius and the metacarpal shaft in pediatric renal transplant recipients compared with controls [21].

## Skeletal structure, strength, and fracture risk

The clinical significance of impaired bone structure lies in the occurrence of fractures. Bone strength reflects the integration of two main features: bone density and bone quality [22]. Bone quality refers to bone architecture, turnover, damage accumulation (e.g. microfractures) and mineralization. CKD likely has adverse effects on each of these components of bone quality. Cortical bone comprises more than 80% of skeletal mass and fulfills a predominantly mechanical function. Cortical bone strength depends on bone geometry, bone density, and the location and direction of applied loads [23]. The long bones are tubular structures that are loaded mainly in bending. The resistance of long bones to bending (i.e. bone strength) is represented by the cross-sectional moment of inertia ${\left( {CSMI} \right)} = \pi  \mathord{\left/  {\vphantom {\pi  4}} \right.  \kern-\nulldelimiterspace} 4{\left( {R^{4}_{p}  - R^{4}_{e} } \right)}$; R_p_ and R_e_ indicate the periosteal and endosteal radius [24]. These power relationships indicate that small decreases in cortical R_p_ or increases in R_e_ markedly decrease bone-bending strength. In addition, cortical vBMD also contributes to bone strength. Ferretti et al. demonstrated that the bone strength index (a measure of CSMI weighted for cortical vBMD) explained 94% of the variability in fracture load in rat femurs [25].

The strength of trabecular bone and its resistance to fracture traditionally have been associated with bone quantity, e.g. bone volume/total volume [BV/TV; or bone volume fraction (BVF)] or apparent density (g/cm^2^ or g/cm^3^). However, trabecular microarchitectural parameters, such as trabecular thickness, number, spacing, orientation (i.e. anisotropy), connectivity, and the ratio of plate- and rod-like structures all contribute to bone strength [26]. For example, a 3D analysis of the spine in autopsy cases in dialysis patients demonstrated normal to increased trabecular BV/TV but abnormal connectivity with frequent and severe trabecular perforations and microcallus formation [27]. Spinal deformities were observed despite normal BV/TV and were attributed to the abnormal trabecular microarchitecture.

## Skeletal modeling during growth and development

The shape and structure of bones is continuously modified and renovated by the two processes of modeling and remodeling. Both result in the replacement of old bone with new bone. Remodeling is the major process in adults and does not result in a change of the bone shape. Remodeling takes place in the basic bone multicellular units on the trabecular surface and within the cortical bone. Normally, bone resorption by osteoclasts is followed by bone formation by osteoblasts; teams of osteoclasts and osteoblasts are juxtaposed in the bone multicellular unit, and bone resorption and formation are tightly coupled. Skeletal remodeling is vital to microdamage repair. However, after midadulthood, the amount of resorption exceeds formation, resulting in a negative bone balance.

In contrast, modeling results in new bone formed at a location different from the site of bone resorption; formation and resorption are not coupled within a bone multicellular unit. Modeling results in an increase in bone diameter and modification of bone shape with linear growth. Growth in the diameter of the cortical shaft is the result of bone formation at the outer (periosteal) surface and bone resorption at the inner (endosteal) surface. Simultaneously, the growth plate moves upward and the wider metaphysis is reshaped into a diaphysis by continuous resorption by osteoclasts beneath the periosteum. The impact of CKD on bone modeling in children has not been characterized.

Cortical and trabecular bone do not respond in the same way to diseases, drugs, growth factors, or sex hormones and should be considered as distinct functional entities. Trabecular vBMD, as measured by spine QCT, does not increase before puberty [28, 29]. During puberty, trabecular vBMD increases significantly due to increases in trabecular thickness. The increases in vBMD are comparable in girls and boys [30] but are significantly greater in black adolescents than in white adolescents [31].

Sex differences in cortical dimensions are established during puberty: cortical width increases by periosteal bone formation in boys and by less periosteal bone formation but more endocortical apposition in girls [32]. Androgens stimulate periosteal apposition whereas estrogens inhibit periosteal apposition and stimulate endosteal apposition. Therefore, boys have a greater cortical CSMI and stronger bones. The timing of CKD relative to pubertal maturation likely has important implications for life-long alterations in bone structure and strength.

## Quantitative assessment of bone status in children and adolescents

DXA is widely accepted as a quantitative measurement technique for assessing skeletal status. In elderly adults, DXA BMD is a sufficiently robust predictor of osteoporotic fractures that it can be used to define the disease. The diagnosis of osteoporosis in adults is based on a T score, the comparison of a measured BMD result with the average BMD of young adults at the time of peak bone mass [33]. Clearly, it is inappropriate to compare a child’s BMD with that of a young adult. Rather, children are assessed relative to age or body size, expressed as a Z score. Despite the growing body of published normative data utilizing DXA in children, there are no evidence-based guidelines for the definition of osteoporosis in children. Fractures occur commonly in otherwise healthy children, with a peak incidence during early adolescence around the time of the pubertal growth spurt [34]. Several studies have compared the DXA BMD of normal children and adolescents with forearm fractures to that of age-matched controls without fractures. Most [35–39], but not all [40, 41], found that mean DXA BMD was significantly lower in children with forearm fractures than in controls.

A significant limitation of DXA is the reliance on measurement of areal rather than volumetric BMD. DXA provides an estimate of BMD expressed as grams per anatomical region (e.g. individual vertebrae, whole body, or hip). Dividing the BMC within the defined anatomical region (g) by the projected area of the bone (cm^2^) then derives “areal BMD” (g/cm^2^). This BMD is not a measure of vBMD (g/cm^3^) because it provides no information about the depth of bone. Bones of larger width and height also tend to be thicker. As bone thickness is not factored into DXA estimates of areal BMD, reliance on DXA inherently underestimates the bone density of short people. Despite identical vBMD, the child with smaller bones appears to have a mineralization disorder (decreased areal BMD) [42]. This is clearly an important artifact in children with chronic diseases, such as CKD, that are associated with poor growth. For this reason, many investigators advocate that areal BMD should not be used in growing children [43–45].

## Limitations of DXA in CKD

**Fig. 1:**
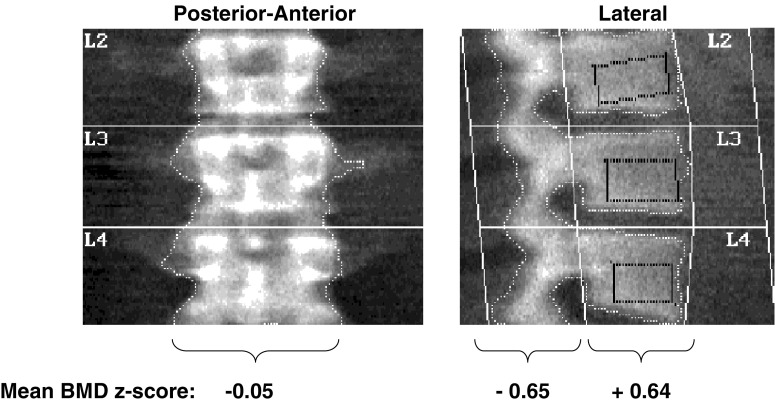
Differential effects of primary hyperparathyroidism on posterior–anterior and lateral dual-energy X-ray absorptiometry (DXA) bone mineral density (BMD) in the spine in adults (data derived from Duan et al. [47]

DXA has additional important limitations in patients with CKD; these limitations were recently reviewed in children undergoing renal transplantation [6]. A study in adults with primary hyperparathyroidism illustrates the limitations of DXA in the setting of increased PTH [46]. Trabecular and cortical bone behave differently in response to increased PTH activity: trabecular bone mass increases and cortical bone mass decreases [1]. The two-dimensional (2D) posterior–anterior DXA projection of the spine captures the largely trabecular vertebral body as well as the superimposed cortical spinous processes. However, the lateral projection allows one to distinguish between the vertebral body and posterior elements. As shown in Fig. 1, mean spine BMD on the posterior–anterior projection was normal; however, the lateral scan revealed increased BMD in the predominantly trabecular vertebral body and decreased BMD in the cortical spinous processes. Consistent with the cortical bone loss in patients with primary hyperparathyroidism, the International Society of Clinical Densitometry (http://www.iscd.org/Visitors/positions) recommends that DXA scans should be performed in the distal one third of the radius (an entirely cortical site) in patients with hyperparathyroidism rather than in the predominantly trabecular lumbar spine or femoral neck.

DXA has been used extensively to evaluate patients with renal osteodystrophy. Clearly, because trabecular and cortical bone behave differently in response to increased parathyroid activity and DXA does not distinguish between renal osteodystrophy effects on the two types of bone, DXA is of limited value in CKD. The conflicting data on DXA-derived measures of BMD in CKD are consistent with these limitations: DXA results in adults have been variable, with mean BMD values higher than, the same as, or lower than control subjects (reviewed in reference [1]). The interpretation of DXA results in children with CKD are further complicated by the confounding effects of poor growth. These difficulties are well-illustrated in the study by Saland et al. [48] in which DXA lumbar spine scans were obtained in 33 children with functional renal allografts an average of 4 years after transplantation. These children had marked growth failure; the mean height Z score was −2.0 + 1.2. The mean DXA BMD Z score relative to gender and chronologic age was −0.9 + 1.3. However, when the BMD Z score was generated relative to gender and height age, the BMD Z score was significantly greater (*p* < 0.001), at 0.4 + 1.4. The difference between the age-specific and height age-specific BMD Z scores were most pronounced in the pubertal transplant recipients. The comparison with controls of the same height age eliminated the confounding effect of bone size; however, this is an incomplete solution, as the renal patients were significantly older than the controls of comparable height. Therefore, the greater BMD in renal patients may represent the effects of greater maturity. Given the extreme short stature of many renal patients, it is not possible to simultaneously adjust for bone size and pubertal maturation.

Early studies of DXA BMD and fracture discrimination yielded conflicting results. Atsumi et al. assessed 187 hemodialysis patients and reported that each standard deviation (SD) decrease in lumbar spine BMD increased the age-adjusted odds ratio (OR) of vertebral fracture 2.0-fold (95% CI: 1.4–2.0) [49]. However, subsequent studies reported that DXA spine BMD provided poor fracture discrimination in dialysis patients and transplant recipients [50–52]. For example, a cross-sectional study of 104 dialysis patients demonstrated comparable spine BMD in the 54 patients with vertebral fractures or fragility fractures compared with those without fractures [51]. Furthermore, the sensitivity and specificity of a DXA spine BMD T score of less than −2.5 (World Health Organization definition of osteoporosis) for the detection of vertebral or fragility fractures were only 25% and 78%, respectively. That is, among subjects with a fracture, 25% had a T score less than −2.5 (sensitivity 25%), and among those without a fracture, 22% had a T score less than −2.5 (specificity 78%). Therefore, the likelihood of a T score less than −2.5 was comparable in patients with and without fracture. The fact that DXA discriminates poorly between fracture and nonfracture cases is not surprising, given that DXA captures poorly the effects of CKD on cortical and trabecular density and quality. I am not aware of any studies relating BMD to fracture risk in children with CKD.

## QCT

**Fig. 2:**
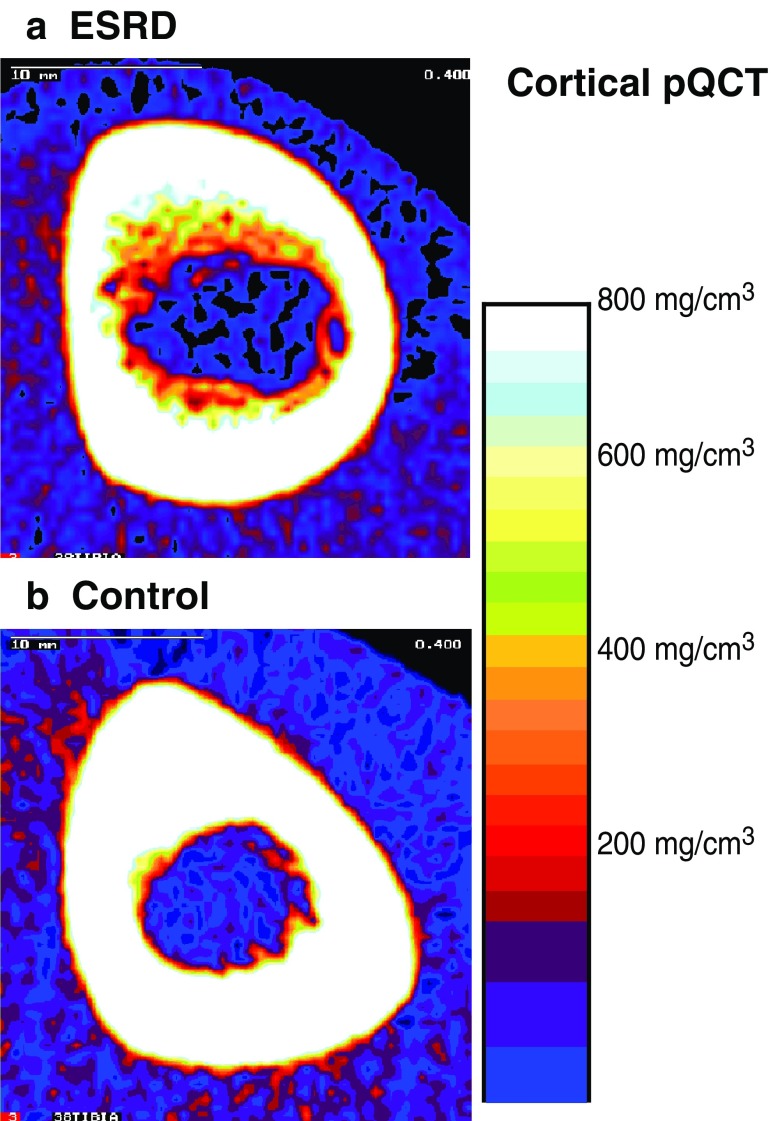
Peripheral skeleton quantitative computed tomography (pQCT) scans in the midshaft of the tibia in a pediatric renal transplant recipient (**a**) and healthy control (**b**). This figure illustrates the bone loss on the endosteal surface and the reduced endocortical volumetric bone mineral density (vBMD)

CT provides a cross-sectional image unobscured by overlying structures [53]. The CT attenuation of different tissues provides quantitative information, referred to as quantitative CT (QCT). In contrast to DXA, this technique describes authentic vBMD (g/cm^3^), accurately measures bone dimensions, and distinguishes between cortical and trabecular bone. In order to minimize radiation exposure, special high-resolution scanners were developed for the peripheral skeleton (pQCT). PQCT can be used to assess trabecular vBMD in the ultradistal radius or tibia and cortical vBMD, periosteal dimensions, and endosteal dimensions in the midshaft. Figure 2 illustrates the endosteal bone loss observed in a 9-year renal transplant recipient at the time of transplantation at our institution, compared with an age, sex, and height-matched control.

Radius pQCT data illustrate the distinct effects of renal osteodystrophy, amenorrhea, and glucocorticoids on cortical and trabecular vBMD (Table 1) [54]. Renal osteodystrophy resulted in increased trabecular vBMD and markedly decreased cortical vBMD, whereas amenorrhea or glucocorticoid therapy resulted in the expected reductions in trabecular vBMD. Spine QCT data in CKD confirmed biopsy histomorphometric data: trabecular vBMD was increased in high-turnover disease (+1.6 SD) and decreased in low-turnover disease (−1.2 SD) relative to age-matched controls [55]. A recent pQCT study in 12 adult transplant recipients demonstrated a significantly greater endosteal circumference and lower cortical thickness compared with controls [56]. Total time on dialysis prior to renal transplantation correlated negatively with cortical thickness (*r* = 0.62; *p* < 0.01).

**Table 1:** Peripheral quantitative computed tomography (pQCT) measures of metabolic bone disease in the radius [54]

	Dialysis	Amenorrhea	Glucocorticoid therapy
Number	10	17	37
Age (years)	39.7±3.7	26.9±6.4	34.1±7.4
Trabecular vBMD	+0.50 SD	−1.49 SD	−0.39 SD
Cortical vBMD	−2.19 SD	−0.76 SD	−0.06 SD

*vBMD* volumetric bone mineral density

A pQCT study in children illustrated the opposing effects of renal osteodystrophy on cortical and trabecular vBMD [20]. PQCT scans and bone biopsies were obtained in 21 children and adolescents treated with maintenance peritoneal dialysis. Trabecular vBMD was significantly greater in CKD subjects compared with controls (*p* < 0.0001), whereas cortical vBMD was significantly lower in CKD subjects compared with controls (*p* < 0.001). Trabecular vBMD was lower in patients with adynamic bone compared with those with high-turnover lesions (*p* < 0.001). In contrast, cortical vBMD was higher in patients with adynamic bone compared with those with high-turnover lesions (*p* < 0.05). Data on cortical thickness or CSMI were not provided. Although pQCT discriminates between trabecular and cortical bone and provides details regarding cortical density and geometry, pQCT is not of sufficient resolution to provide detailed microarchitectural information in the trabecular sites. This limitation is illustrated in the study below relating pQCT measures to fracture risk.

## DXA and pQCT receiver operating characteristic curves in CKD

The diagnostic test characteristics of the varied imaging modalities in discriminating fracture risk is best assessed using receiver-operating characteristic (ROC) curves [57, 58]. To assess the discriminative ability of the varied tests, ROC curves can be constructed for each scan outcome; e.g. DXA areal-BMD, pQCT cortical thickness, or pQCT trabecular vBMD. ROC curves plot the sensitivity on the y-axis versus 1-specificity on the x-axis. A variable that has excellent predictive power for fracture will tend, at all possible cutoff values, to have greater sensitivity (and hence be farther “up”) and greater specificity (and hence be farther to the left), than a test with worse predictive value. The predictive power is assessed by computing the area under the ROC curve, or the AUC; as the AUC approaches 1.0, the greater the predictive power of the test. To compare measures directly (e.g. to test the hypothesis that pQCT measures of cortical vBMD provide better fracture discrimination than DXA areal BMD), one can test the equality of the AUC values using a χ^2^ test.

**Fig. 3:**
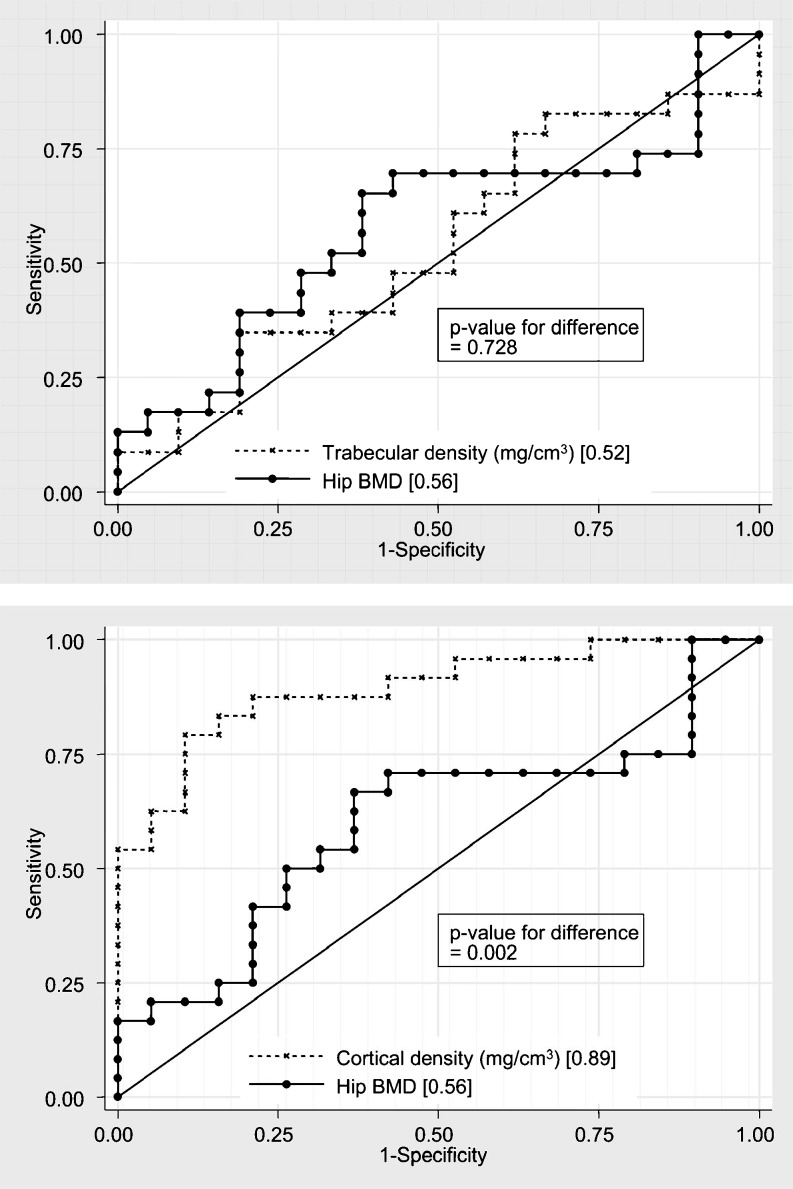
Receiver operating characteristic (ROC) curves for dual-energy X-ray absorptiometry (DXA) areal bone mineral density (BMD) in the hip and peripheral skeleton quantitative computed tomography (pQCT) cortical volumetric BMD (vBMD) in the midshaft of the radius in dialysis patients (From Jamal et al. [59])

A recent cross-sectional ROC study in 52 adults on maintenance hemodialysis demonstrated that reductions in pQCT measures of cortical bone in the radius were strongly predictive of vertebral and fragility fractures, whereas pQCT trabecular vBMD and DXA areal BMD were not [59]. Table 2 illustrates the marked differences in the odds of fracture and AUC at each site. For example, the AUC for DXA areal BMD in the hip was only 0.56, whereas the AUC for pQCT cortical vBMD was 0.89; this difference was significant, as illustrated in Fig. 3. The clinical significance of these marked differences is highlighted by the OR in Table 2. Of note, this study did not include DXA measures in the radius. Therefore, it is not known if DXA measures in the cortical midshaft of the radius will improve fracture discrimination. Finally, the failure of pQCT trabecular vBMD to discriminate fractures in dialysis patients is not surprising, given that pQCT does not have sufficient resolution to detect the unique effects of renal osteodystrophy on trabecular microarchitecture independent of bone quantity. Recall that a 3D analysis of the spine in autopsy cases in dialysis patients demonstrated that abnormal connectivity was associated with vertebral deformities despite normal to increased BV/TV [27].

**Table 2:** Association between fractures and peripheral skeleton quantitative computed tomography (pQCT) or dual-energy X-ray absorptiometry (DXA) measures in adult hemodialysis patients [59]

Predictor	Odds of fracture (95% CI) per SD decrease	Area under ROC curve
pQCT		
Trabecular vBMD	1.18 (0.60, 2.33)	0.52 (0.34, 0.39)
Cortical vBMD	16.67 (2.94, 83.3)	0.89 (0.90, 0.99)
Cortical Thickness	3.26 (1.36, 7.87)	0.78 (0.63, 0.93)
DXA		
Spine areal BMD	0.53 (0.27, 1.06)	0.63 (0.48, 0.78)
Femoral neck areal BMD	0.69 (0.36, 1.30)	0.42 (0.23, 0.57)
Total hip areal BMD	1.10 (0.85, 2.08)	0.56 (0.30, 0.63)

*vBMD* volumetric bone mineral density, *BMD* bone mineral density

## Research applications: micro-CT and micro-MRI

**Fig. 4:**
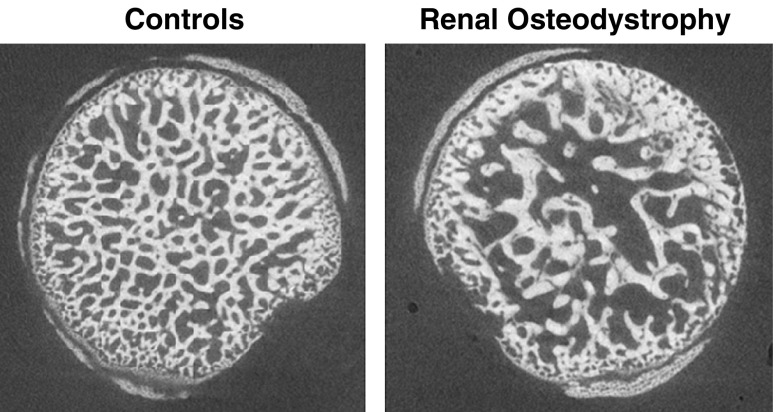
Axial micro computed tomography (μCT) images (8.2-μm resolution) in the femoral neck illustrate the effects of renal osteodystrophy on trabecular microarchitecture in growing rats. (Adapted from Hopper et al. [60])

The newest generation of high-resolution CT and MRI scanners can be used to evaluate bone microarchitecture. For example, we recently used μCT to assess the structure effects of severe high-turnover renal osteodystrophy on trabecular and cortical architecture in growing rats [60]. Figure 4 illustrates the irregular trabecular thickening and loss of trabecular connectivity in rats with severe secondary hyperparathyroidism. Cortical scans revealed significantly greater endocortical porosity in the rats with renal osteodystrophy compared with controls. Note that these scans were obtained at 8-μm resolution (Fig. 4) and scan acquisition required greater than 3 h; therefore, this technique is limited to in vitro applications.

Micro-MRI can be performed in vivo and has been used to illustrate that age-related and postmenopausal bone loss proceeds via inhomogeneous thinning of trabecular plates, leading to fenestration of plates and conversion to rods. These rods may become disconnected and eventually resorbed entirely. Recent advances in μMRI, in conjunction with image processing and feature extraction processes, allow detailed structural information to be obtained from the μMRI “virtual bone biopsy” [61]. In addition to morphologic parameters synonymous to standard histomorphometry [e.g. BV/TV, trabecular separation (Tb.Sp), trabecular thickness (Tb.Th) and trabecular number (Tb.N)], digital topologic analysis of μMRIs generates estimates of trabecular connectivity and the conversion of plates to rods [62]. Recent studies demonstrate that these measures improve fracture prediction and are sensitive to structure changes induced by testosterone replacement in hypogonadal men [63] and hormone replacement therapy in women [64].

**Fig. 5:**
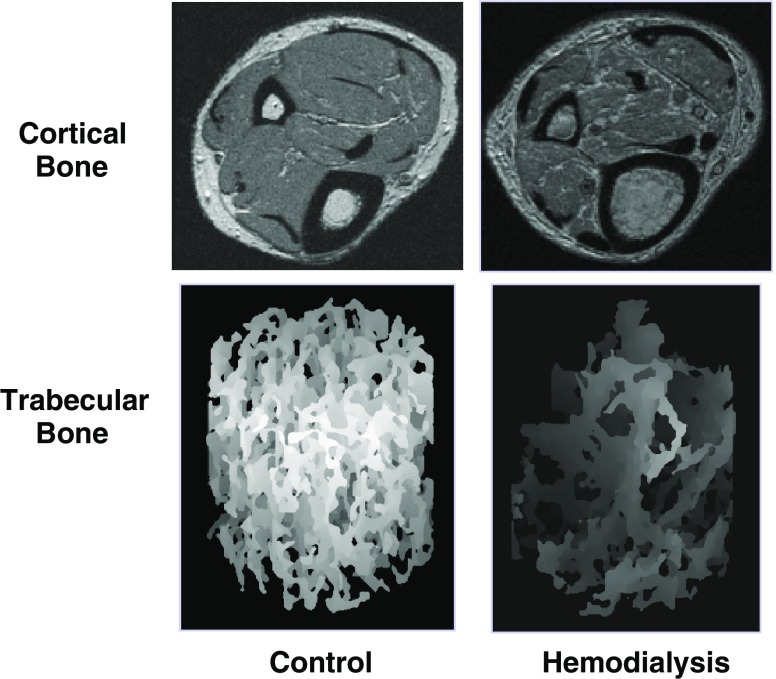
Tibia micro magnetic resonance imaging (μMRI) illustrates severe cortical thinning and loss of trabecular connectivity and bone volume in a hemodialysis patient compared with a healthy age- and gender-matched control (Adapted from Wehrli et al. [65])

We recently completed a pilot study of μMRI in 17 young adults on maintenance hemodialysis compared with healthy controls [65]. Figure 5 illustrates the cortical thinning and disruption of trabecular architecture in a young adult male with severe uncontrolled renal osteodystrophy of many years duration, ultimately resulting in parathyroidectomy. BV/TV did not differ significantly in this small sample of hemodialysis patients compared with controls; however, the pattern of lower surface and greater curve voxels suggested deterioration of the trabecular network.

Two cross-sectional studies used high-resolution CT [66] or MRI [67] to evaluate trabecular architecture in renal transplant recipients; patients were evaluated months to years after transplantation. Grotz et al. reported that vertebral deformities were associated with lower trabecular area, lower trabecular diameter, and increased intertrabecular spacing in transplant recipients compared with transplant recipients without vertebral deformities [66]. Link et al. reported that transplant recipients had significantly lower MRI measures of BV/TV, Tb.Sp, Tb.Th, and Tb.N compared with controls; [67] however, spine QCT, trabecular BMD, and DXA hip and spine BMD did not differ between transplant recipients and controls. The MRI and QCT measures differed between fracture and nonfracture end-stage renal disease (ESRD) patients; DXA measures did not.

## Summary

The 2003 National Kidney Foundation Clinical Practice Guidelines for Bone Metabolism and Disease in CKD recommended the following for all adult patients with CKD: [68] “Bone mineral density should be measured by dual energy X-ray absorptiometry in patients with fractures and in those with known risk factors for osteoporosis.” These guidelines did not specify the measurement site. The pediatric guidelines did not recommend DXA in children, given the limitations in CKD and the lack of consensus on the optimal approach to adjust for the confounding effect of bone size during growth [69]. More recently, the Kidney Disease: Improving Global Outcomes working group published a position paper of the evaluation of renal osteodystrophy [70]. The report concluded that the value of DXA BMD measurement in the evaluation of CKD metabolic bone disease is not well established and concluded that the radius is the preferred site of measurement in CKD patients, consistent with the recommendations of the International Society of Clinical Densitometry, as detailed above.

The assessment of the effects of renal osteodystrophy and its potential therapies on fracture risk requires novel imaging techniques to characterize accurately the effects on trabecular and cortical bone. Recent data suggest that DXA has minimal, if any, utility in predicating fractures in advanced CKD. Valid surrogate measures of fracture risk (e.g. QCT measures of cortical vBMD or μMRI measures of trabecular connectivity) are required for clinical trials of therapeutic agents. Ongoing research in this field will guide future studies to improve bone strength and decrease fracture rates in a population that will continue to require renal replacement therapies throughout their lifetime.

## References

[1] Parfitt AM (1998). A structural approach to renal bone disease. J Bone Miner Res.

[2] Alem AM, Sherrard DJ, Gillen DL, Gillen DL, Weiss NS, Beresford SA, Heckbert SR, Wong C, Stelunan-Breen C (2000). Increased risk of hip fracture among patients with end-stage renal disease. Kidney Int.

[3] Ball AM, Gillen DL, Sherrard D, Weiss NS, Emerson SS, Seliger SL, Kestenbaum BR, Stelunan-Breen C (2002). Risk of hip fracture among dialysis and renal transplant recipients. JAMA.

[4] Helenius I, Remes V, Salminen S, Valtra H, Makitie O, Holmberg C, Palmu P, Tervahartiala P, Sarna S, Helnius M, Peltonen J, Jalanko H (2006). Incidence and predictors of fractures in children after solid organ transplantation: a 5-year prospective, population-based study. J Bone Miner Res.

[5] Helenius I, Remes V, Tervahartiala P, Salminen S, Sairanen H, Holmberg C, Palmu P, Helenius M, Peltonen J, Jalanko H (2006). Spine after solid organ transplantation in childhood: a clinical, radiographic, and magnetic resonance imaging analysis of 40 patients. Spine.

[6] Leonard MB (2005). Assessment of bone mass following renal transplantation in children. Pediatr Nephrol.

[7] Schonau E (1998). Problems of bone analysis in childhood and adolescence. Pediatr Nephrol.

[8] Chesney RW, Moorthy AV, Eisman JA, Jax DK, Mazess RB, DeLuca HF (1978). Increased growth after long-term oral 1alpha,25-vitamin D3 in childhood renal osteodystrophy. N Engl J Med.

[9] Salusky IB, Coburn JW, Brill J, Foley J, Slatopolsky F, Fine RN, Goodman WG (1988). Bone disease in pediatric patients undergoing dialysis with CAPD or CCPD. Kidney Int.

[10] Salusky IB, Ramirez JA, Oppenheim W, Gales B, Segre GV, Goodman WG (1994). Biochemical markers of renal osteodystrophy in pediatric patients undergoing CAPD/CCPD. Kidney Int.

[11] Ziolkowska H, Paniczyk-Tomaszewska M, Debinski A, Polowiec Z, Sawicki A, Sieniawska M (2000). Bone biopsy results and serum bone turnover parameters in uremic children. Acta Paediatr.

[12] Yalcinkaya F, Ince E, Tumer N, Ensari A, Ozkaya N (2000). Spectrum of renal osteodystrophy in children on continuous ambulatory peritoneal dialysis. Pediatr Int.

[13] Salusky IB, Kuizon BD, Belin TR, Ramirez JA, Gales B, Segre GV, Goodman WG (1998). Intermittent calcitriol therapy in secondary hyperparathyroidism: a comparison between oral and intraperitoneal administration. Kidney Int.

[14] Coco M, Rush H (2000). Increased incidence of hip fractures in dialysis patients with low serum parathyroid hormone. Am J Kidney Dis.

[15] Kuizon BD, Goodman WG, Juppner H, Boechat I, Nelson P, Gales B, Salusky IB (1998). Diminished linear growth during intermittent calcitriol therapy in children undergoing CCPD. Kidney Int.

[16] Schmitt CP, Ardissino G, Testa S, Claris-Appiani A, Mehls O (2003). Growth in children with chronic renal failure on intermittent versus daily calcitriol. Pediatr Nephrol.

[17] Waller S, Ledermann S, Trompeter R, van’t Hoff W, Ridout D, Rees L (2003). Catch-up growth with normal parathyroid hormone levels in chronic renal failure. Pediatr Nephrol.

[18] Hruska KA, Teitelbaum SL (1995). Renal osteodystrophy. N Engl J Med.

[19] Schober HC, Han ZH, Foldes AJ, Shih MS, Rao DS, Balena R, Parfitt AM (1998). Mineralized bone loss at different sites in dialysis patients: implications for prevention. J Am Soc Nephrol.

[20] Lima EM, Goodman WG, Kuizon BD, Gales B, Emerick A, Goldin J, Salusky IB (2003). Bone density measurements in pediatric patients with renal osteodystrophy. Pediatr Nephrol.

[21] Ruth EM, Weber LT, Schoenau E, Wunsch R, Seibel MJ, Feneberg R, Mehls O, Tonshoff B (2004). Analysis of the functional muscle-bone unit of the forearm in pediatric renal transplant recipients. Kidney Int.

[22] NIH (2000). Osteoporosis prevention, diagnosis, and therapy. NIH Consens Statement.

[23] Turner CH (1993). Basic biomechanical measurements of bone: a tutorial. Bone.

[24] Burr DB, Turner CH, Flavus MJ (2003). Biomechanics of bone. Primer on the metabolic bone diseases and disorders of mineral metabolism.

[25] Ferretti JL (1995). Perspectives of pQCT technology associated to biomechanical studies in skeletal research employing rat models. Bone.

[26] Mittra E, Rubin C, Qin YX (2005). Interrelationship of trabecular mechanical and microstructural properties in sheep trabecular bone. J Biomech.

[27] Amling M, Grote HJ, Vogel M, Hahn M, Delling G (1994). Three-dimensional analysis of the spine in autopsy cases with renal osteodystrophy. Kidney Int.

[28] Gilsanz V, Roe TF, Mora S, Costin G, Goodman WG (1991). Changes in vertebral bone density in black girls and white girls during childhood and puberty. N Engl J Med.

[29] Gilsanz V, Kovanlikaya A, Costin G, Roe TF, Sayre J, Kaufman F (1997). Differential effect of gender on the sizes of the bones in the axial and appendicular skeletons. J Clin Endocrinol Metab.

[30] Gilsanz V, Gibbens DT, Roe TF, Carlson M, Senac MO, Boechat MI, Huang HK, Schulz FF, Libanati CR, Cann CC (1988). Vertebral bone density in children: effect of puberty. Radiology.

[31] Han ZH, Palnitkar S, Rao DS, Nelson D, Parfitt AM (1996). Effect of ethnicity and age or menopause on the structure and geometry of iliac bone. J Bone Miner Res.

[32] Seeman E (2002). Pathogenesis of bone fragility in women and men. Lancet.

[33] WHO (1994) The WHO Study Group: assessment of fracture risk and its application to screening for postmenopausal osteoporosis. Geneva, Switzerland 7941614

[34] Khosla S, Melton LJ, Dekutoski MB, Achenbach SJ, Oberg AL, Riggs BL (2003). Incidence of childhood distal forearm fractures over 30 years: a population-based study. JAMA.

[35] Chan GM, Hess M, Hollis J, Book LS (1984). Bone mineral status in childhood accidental fractures. Am J Dis Child.

[36] Goulding A, Cannan R, Williams SM, Gold EJ, Taylor RW, Lewis-Barned NJ (1998). Bone mineral density in girls with forearm fractures. J Bone Miner Res.

[37] Goulding A, Jones IE, Taylor RW, Williams SM, Manning PJ (2001). Bone mineral density and body composition in boys with distal forearm fractures: a dual-energy X-ray absorptiometry study. J Pediatr.

[38] Goulding A, Jones IE, Taylor RW, Manning PJ, Williams SM (2000). More broken bones: a 4-year double cohort study of young girls with and without distal forearm fractures. J Bone Miner Res.

[39] Ma D, Jones G (2003). The association between bone mineral density, metacarpal morphometry, and upper limb fractures in children: a population-based case-control study. J Clin Endocrinol Metab.

[40] Ma DQ, Jones G (2002). Clinical risk factors but not bone density are associated with prevalent fractures in prepubertal children. J Paediatr Child Health.

[41] Cook SD, Harding AF, Morgan EL, Doucet JH, Bennett JT, O’Brien M, Thomas KA (1987). Association of bone mineral density and pediatric fractures. J Pediatr Orthop.

[42] Wren TA, Liu X, Pitukcheewanont P, Gilsanz V (2005). Bone densitometry in pediatric populations: discrepancies in the diagnosis of osteoporosis by DXA and CT. J Pediatr.

[43] Chauhan S, Koo WW, Hammami M, Hockman EM (2003). Fan beam dual energy X-ray absorptiometry body composition measurements in piglets. J Am Coll Nutr.

[44] Nelson DA, Koo WW (1999). Interpretation of absorptiometric bone mass measurements in the growing skeleton: issues and limitations. Calcif Tissue Int.

[45] Rauch F, Schoenau E (2001). Changes in bone density during childhood and adolescence: an approach based on bone’s biological organization. J Bone Miner Res.

[46] Miller MA, Chin J, Miller SC, Fox J (1998). Disparate effects of mild, moderate, and severe secondary hyperparathyroidism on cancellous and cortical bone in rats with chronic renal insufficiency. Bone.

[47] Duan Y, De Luca V, Seeman E (1999). Parathyroid hormone deficiency and excess: similar effects on trabecular bone but differing effects on cortical bone. J Clin Endocrinol Metab.

[48] Saland JM, Goode ML, Haas DL, Romano TA, Seikaly MG (2001). The prevalence of osteopenia in pediatric renal allograft recipients varies with the method of analysis. Am J Transplant.

[49] Atsumi K, Kushida K, Yamazaki K, Shimizu S, Ohmura A, Inoue T (1999). Risk factors for vertebral fractures in renal osteodystrophy. Am J Kidney Dis.

[50] Yamaguchi T, Kanno E, Tsubota J, Shiomi T, Nakai M, Hattori S (1996). Retrospective study on the usefulness of radius and lumbar bone density in the separation of hemodialysis patients with fractures from those without fractures. Bone.

[51] Jamal SA, Chase C, Goh YI, Richardson R, Hawker GA (2002). Bone density and heel ultrasound testing do not identify patients with dialysis-dependent renal failure who have had fractures. Am J Kidney Dis.

[52] Grotz WH, Mundinger FA, Gugel B, Exner V, Kirste G, Schollmeyer PJ (1994). Bone fracture and osteodensitometry with dual energy X-ray absorptiometry in kidney transplant recipients. Transplantation.

[53] Gilsanz V (1998). Bone density in children: a review of the available techniques and indications. Eur J Radiol.

[54] Tsurusaki K, Ito M, Hayashi K (2000). Differential effects of menopause and metabolic disease on trabecular and cortical bone assessed by peripheral quantitative computed tomography (pQCT). Br J Radiol.

[55] Torres A, Lorenzo V, Gonzalez-Posada JM (1986). Comparison of histomorphometry and computerized tomography of the spine in quantitating trabecular bone in renal osteodystrophy. Nephron.

[56] Negri AL, Lombas C, Cuevas C, Schiavelli R, Bogado CE, Zanchetta JR (2005). Evaluation of cortical bone by peripheral quantitative computed tomography in renal transplant recipients. Transplant Proc.

[57] Hanley JA, McNeil BJ (1983). A method of comparing the areas under receiver operating characteristic curves derived from the same cases. Radiology.

[58] DeLong ER, DeLong DM, Clarke-Pearson DL (1988). Comparing the areas under two or more correlated receiver operating characteristic curves: a nonparametric approach. Biometrics.

[59] Jamal SA, Gilbert J, Gordon C, Bauer DC (2006). Cortical pQCT measures are associated with fractures in dialysis patients. J Bone Miner Res.

[60] Hopper TAJ, Wehrli FW, Andre JB, Wright AC, Sanchez CP (2007). Quantitative micro CT assessment of intra- and inter-trabecular and cortical bone architecture in a model of advanced renal osteodystrophy in a growing rat. J Comput Tomogr.

[61] Wehrli FW, Saha PK, Gomberg BR, Song HK (2003). Noninvasive assessment of bone architecture by magnetic resonance micro-imaging-based virtual bone biopsy. Proceedings IEEE.

[62] Wehrli FW, Gomberg BR, Saha PK, Song HK, Hwang SN, Snyder PJ (2001). Digital topological analysis of in vivo magnetic resonance microimages of trabecular bone reveals structural implications of osteoporosis. J Bone Miner Res.

[63] Benito M, Vasilic B, Wehrli FW, Bunker B, Wald M, Gomberg B, Wright AC, Zemel B, Cucchiara A, Snyder PJ (2005). Effect of testosterone replacement on trabecular architecture in hypogonadal men. J Bone Miner Res.

[64] Ladinsky GA, Vasilic B, Popescu AM, Zemel BS, Wright AC, Song HK, Saha PK, Peachy H, Synder PJ, Wehrli FW (2005). MRI based virtual bone biopsy detects large one-year changes in trabecular bone architecture of early postmenopausal women. J Bone Min Res.

[65] Wehrli FW, Leonard MB, Saha PK, Gomberg BR (2004). Quantitative high-resolution magnetic resonance imaging reveals structural implications of renal osteodystrophy on trabecular and cortical bone. J Magn Reson Imaging.

[66] Grotz WH, Mundinger FA, Muller CB, Rasenack J, Schulte-Monting J, Langer MF, Schollmeyer PJ (1997). Trabecular bone architecture in female renal allograft recipients-assessed by computed tomography. Nephrol Dial Transplant.

[67] Link TM, Saborowski, Kisters K, Kempkes M, Kosch M, Newitt D, Lu Y, Waldt S, Majuindar S (2002). Changes in calcaneal trabecular bone structure assessed with high-resolution MR imaging in patients with kidney transplantation. Osteoporos Int.

[68] K/DOQI (2003). Clinical practice guidelines for bone metabolism and disease in chronic kidney disease. Am J Kidney Dis.

[69] K/DOQI (2005). Clinical Practice Guidelines for bone metabolism and disease in children with chronic kidney disease. Am J Kidney Dis.

[70] Moe S, Drueke T, Cunningham J, Goodman W, Martin K, Olgaard K, Ott S, Sprague S, Lameire N, Eknoyan G (2006). Definition, evaluation, and classification of renal osteodystrophy: a position statement from kidney disease: improving global outcomes (KDIGO). Kidney Int.

